# The PDE4 Inhibitor Tanimilast Blunts Proinflammatory Dendritic Cell Activation by SARS-CoV-2 ssRNAs

**DOI:** 10.3389/fimmu.2021.797390

**Published:** 2022-01-24

**Authors:** Hoang Oanh Nguyen, Tiziana Schioppa, Laura Tiberio, Fabrizio Facchinetti, Gino Villetti, Maurizio Civelli, Annalisa Del Prete, Francesca Sozio, Carolina Gaudenzi, Mauro Passari, Ilaria Barbazza, Silvano Sozzani, Valentina Salvi, Daniela Bosisio

**Affiliations:** ^1^ Department of Molecular and Translational Medicine, University of Brescia, Brescia, Italy; ^2^ IRCCS Humanitas Research Hospital, Rozzano, Italy; ^3^ Chiesi Farmaceutici S.p.A., Corporate Pre-Clinical R&D, Parma, Italy; ^4^ Laboratory Affiliated to Istituto Pasteur Italia-Fondazione Cenci Bolognetti, Department of Molecular Medicine, Sapienza University of Rome, Rome, Italy; ^5^ IRCCS Neuromed, Pozzilli, Italy

**Keywords:** COVID-19, proinflammatory cytokines, cDCs, pDCs, phosphodiesterase 4 (PDE4) inhibitors

## Abstract

Phosphodiesterase 4 (PDE4) inhibitors are immunomodulatory drugs approved to treat diseases associated with chronic inflammatory conditions, such as COPD, psoriasis and atopic dermatitis. Tanimilast (international non-proprietary name of CHF6001) is a novel, potent and selective inhaled PDE4 inhibitor in advanced clinical development for the treatment of COPD. To begin testing its potential in limiting hyperinflammation and immune dysregulation associated to SARS-CoV-2 infection, we took advantage of an *in vitro* model of dendritic cell (DC) activation by SARS-CoV-2 genomic ssRNA (SCV2-RNA). In this context, Tanimilast decreased the release of pro-inflammatory cytokines (TNF-α and IL-6), chemokines (CCL3, CXCL9, and CXCL10) and of Th1-polarizing cytokines (IL-12, type I IFNs). In contrast to β-methasone, a reference steroid anti-inflammatory drug, Tanimilast did not impair the acquisition of the maturation markers CD83, CD86 and MHC-II, nor that of the lymph node homing receptor CCR7. Consistent with this, Tanimilast did not reduce the capability of SCV2-RNA-stimulated DCs to activate CD4^+^ T cells but skewed their polarization towards a Th2 phenotype. Both Tanimilast and β-methasone blocked the increase of MHC-I molecules in SCV2-RNA-activated DCs and restrained the proliferation and activation of cytotoxic CD8^+^ T cells. Our results indicate that Tanimilast can modulate the SCV2-RNA-induced pro-inflammatory and Th1-polarizing potential of DCs, crucial regulators of both the inflammatory and immune response. Given also the remarkable safety demonstrated by Tanimilast, up to now, in clinical studies, we propose this inhaled PDE4 inhibitor as a promising immunomodulatory drug in the scenario of COVID-19.

## Introduction

SARS coronavirus 2 (SARS-CoV-2), the causative agent of the pandemic Coronavirus disease 2019 (COVID-19), is a positive-sense ssRNA virus belonging to the family of Coronaviridae (1). In a subgroup of patients, COVID-19 develops as acute respiratory distress syndrome (ARDS) featuring intense lung injury, sepsis-like manifestations and multi-organ failure (2). Dysfunctional immune response and hyper-inflammation with subsequent cytokine storm were shown to play a key role in the development of severe and fatal forms of COVID-19 (3). We recently described a novel mechanism of SARS-CoV-2-dependent activation of innate immune cells, based on the recognition of sequences of viral genomic ssRNA (SCV2-RNA) by endosomal pattern recognition receptors, namely TLR7 and TLR8 (4). Of note, SCV2-RNA recapitulated potent lung inflammation *in vivo* and induced a strong release of pro-inflammatory cytokines and Th1 polarization *in vitro*.

Several immunomodulatory therapies targeting the inflammation-driven damaging stages were proposed for the treatment of severe COVID-19 (5). Among these, inhibitors of phosphodiesterases (PDEs) have been put forward based on the analogy between the clinical features of COVID-19 and other pathologies, associated with inflammation, for which these drugs are already approved (6). PDEs are a superfamily of 11 isoenzymes that modulate signal transduction by degrading cyclic nucleotides (cAMP and/or cGMP). PDE4s, comprising PDE4A, PDE4B, PDE4C and PDE4D, are cAMP-specific PDEs abundantly expressed in leukocytes (7), where they promote the production of pro-inflammatory cytokines and lipid mediators (8). Inhibition of PDE4 leads to accumulation of intracellular cAMP and to a shift of the anti-inflammatory/pro-inflammatory balance (8). Such upstream anti-inflammatory mechanism, makes these agents particularly interesting to master critical conditions characterized by overt release of multiple cytokines, as compared to other single downstream anti-cytokine drugs (9). Nevertheless, side effects such as gastrointestinal disturbances, particularly nausea and emesis as well as headache and weight loss are typically associated with oral PDE4 inhibitors (10). Tanimilast (international non-proprietary name of CHF6001) is an inhaled, selective inhibitor of PDE4 isoforms A-D (11) endowed with anti-inflammatory properties in several *in vitro* and *in vivo* models (12, 13) which is particularly well tolerated as compared to oral PDE4 inhibitors (13) given its high lung retention coupled with low systemic exposure (14). Published data by our group highlighted that Tanimilast can reduce the secretion of inflammatory and Th1/Th17 polarizing cytokines by fine tuning the activity of the master inflammatory transcription factor NF-κB, which could be useful to control Th-1 and Th-17 driven pathologies without inducing a global repression of the inflammatory and immune responses (15).

Dendritic cells (DCs) are innate immune cells that, by expressing several nucleic acid sensors, play a crucial role in recognizing viral pathogens and mounting protective inflammatory and interferon responses. In addition, DCs are specialized antigen presenting cells capable of activating and shaping the adaptive response, both CD4^+^ and CD8^+^ T cell-mediated, to clear the infection (16). Given the central role of DCs in the regulation of the immune response, excessive activation of these cells may unleash overt immunity and tissue damage (16, 17). During the progression of SARS-CoV-2 infection, both DCs and CD4^+^ and CD8^+^ T cell are recruited to the lung (18, 19), with Th1/Th17 effectors reported to play a pivotal role in severe COVID-19 pneumonia (20, 21). Thus, DCs represent an interesting pharmacological target to modulate detrimental immune responses, possibly including those observed in severe forms of COVID-19.

This study was designed to investigate the effects of Tanimilast on DC activation induced by SCV2-RNA with the aim of uncovering the potential beneficial immunomodulatory effects of such drug in COVID-19.

## Materials and Methods

### Cell Preparation and Culture

Buffy coats from blood donations of anonymous healthy donors were obtained and preserved by the Centro Trasfusionale, Spedali Civili of Brescia according to the Italian law concerning blood component preparation and analysis. Peripheral blood mononuclear cells (PBMC) were obtained by density gradient centrifugation and monocytes were subsequently purified by immunomagnetic separation using anti CD14-conjugated magnetic microbeads (Miltenyi Biotec) according to the manufacture’s protocol and as previously published (22). Briefly, monocytes were cultured for 6 days in tissue culture plates in complete medium (RPMI 1640 supplemented with 10% heat-inactivated, endotoxin free FBS, 2 mM L-Glutamine, penicillin and streptomycin (all from Gibco, Thermo Fisher Scientific) in the presence of 50 ng/ml GM-CSF and 20 ng/ml IL-4 (Miltenyi Biotec). Untouched peripheral blood cDC1 and cDC2 (cDCs) and pDCs were obtained from PBMC after negative immunomagnetic separation with the Myeloid Dendritic Cell Isolation kit (Miltenyi Biotec) and the Plasmacytoid Dendritic Cell Isolation kit II (Miltenyi Biotec), respectively.

### Cell Stimulation With SCV2-RNA

Complexation of SCV2-RNA (5’-UGCUGUUGUGUGUUU-3’; genome position: 15692-15706) with DOTAP Liposomal Transfection Reagent (Roche) was performed as previously described (4). Briefly, 5 μg RNA in 50 μl HBS buffer (20 mM HEPES, 150 mM NaCl, pH 7.4) was combined with 100 μl DOTAP solution (30 μl DOTAP plus 70 μl HBS buffer) and incubated for 15 minutes at RT. After the complexation, 150 μl of HBS was added and used to stimulate the cells (final concentration of 5 μg/ml). Where indicated, cells (2x10^6^/ml in 48 well-plate) were pretreated for 1 hour with the indicated concentrations of Tanimilast or β-methasone (provided by Chiesi Farmaceutici S.p.A.). The maturation process was conducted in RPMI containing 2% FBS and supplemented with 0.01% DMSO to avoid the sequestration of Tanimilast by serum proteins.

### Cytokine Detection

TNF-α, IL-1β, IL-6, IL-12p70, CXCL8, CXCL10, CCL3, CCL17 were measured by ELISA assay (R&D Systems). IFN-α was detected using specific Module Set ELISA kit (eBioscience) and IFN-β by was measured by a bioluminescence kit (*In vivo*Gen). All assays were performed on cell free supernatants according to the manufacturer’s protocol.

### Flow Cytometry

DCs were stained with the following antibodies from Miltenyi Biotec: Vioblue-conjugated anti-human CD86 (clone FM95), PE-conjugated anti-human CD83 (clone REA714), FITC-conjugated anti-human BDCA2 (clone AC144), APC-conjugated anti-human CCR7 (clone REA546). Cell viability was assessed by LIVE/DEAD staining according to the manufacturer’s instruction (Molecular Probes, Thermo Fisher Scientific). Samples were read on a MACSQuant Analyzer (Miltenyi Biotec) and analysed with FlowJo (Tree Star Inc.). Response definition criteria were defined *post-hoc*. Raw data can be provided per request.

### T Cell Proliferation Assay

Because Tanimilast was previously shown to exert direct effects on T lymphocytes (23, 24), stimulated moDCs were collected and thoroughly washed to avoid any Tanimilast contamination of cocultures. Allogenic naïve CD4^+^ T cells and CD8^+^ T cells were isolated from buffycoats using the naïve CD4^+^ T cell Isolation kit II (Miltenyi Biotec) and CD8^+^ T cell Isolation kit (Miltenyi Biotec), respectively. Purified T cells were labeled with CellTrace-CFSE (Molecular Probes, Thermo Fisher Scientific) at a final concentration of 5 μM. Subsequently, T cells (6x10^4^ cells/well) were co-cultured with graded numbers of moDCs in 96-well round-bottom culture plates in complete RPMI medium. After 6 days, alloreactive T cell proliferation was assessed by measuring the loss of the dye CellTrace-CFSE upon cell division using flow cytometry. Positive controls of T cell proliferations were routinely performed using IL-2 plus PHA. Response definition criteria were defined *post-hoc*. Dead cells were excluded by LIVE/DEAD staining according to the manufacturer’s instruction. Raw data can be provided per request.

### Analysis of T Cell Cytokine Production

After 6 days of co-culture, CD4^+^ and CD8^+^ T cells were restimulated with 200 nM PMA (Sigma-Aldrich) plus 1 μg/ml of ionomycin (Sigma) for 4.5 hours. Brefeldin A (5 μg/ml, Sigma) was added during the last 2 hours. For intracellular cytokine production, cells were fixed and permeabilized with Inside Stain kit (Miltenyi Biotec) and stained with FITC-conjugated anti-IFN-γ (clone 45-15, Miltenyi Biotec), PE-conjugated anti-IL-4 (clone 7A3-3, Miltenyi Biotec), APC-conjugated anti-IL-13 (clone OES10-5A2, Biolegend), APC-conjugated anti GrB (clone REA226) following the manufacturer’s recommendations. Response definition criteria were defined *post-hoc*. Raw data can be provided per request.

### Statistical Analysis

Sample group normality was confirmed by Shapiro-Wilk test before application of parametric statistical analysis. Statistical significance among the experimental groups was determined using one-way ANOVA with Dunnet’s *post-hoc* test (GraphPad Prism 7, GraphPad Software) as indicated in each figure legend. P< 0.05 was considered significant.

## Results

### Tanimilast Selectively Reduces the Secretion of Cytokines and Chemokines by moDCs Stimulated With SCV2-RNA (SCV2-moDCs)

**Figure 1:**
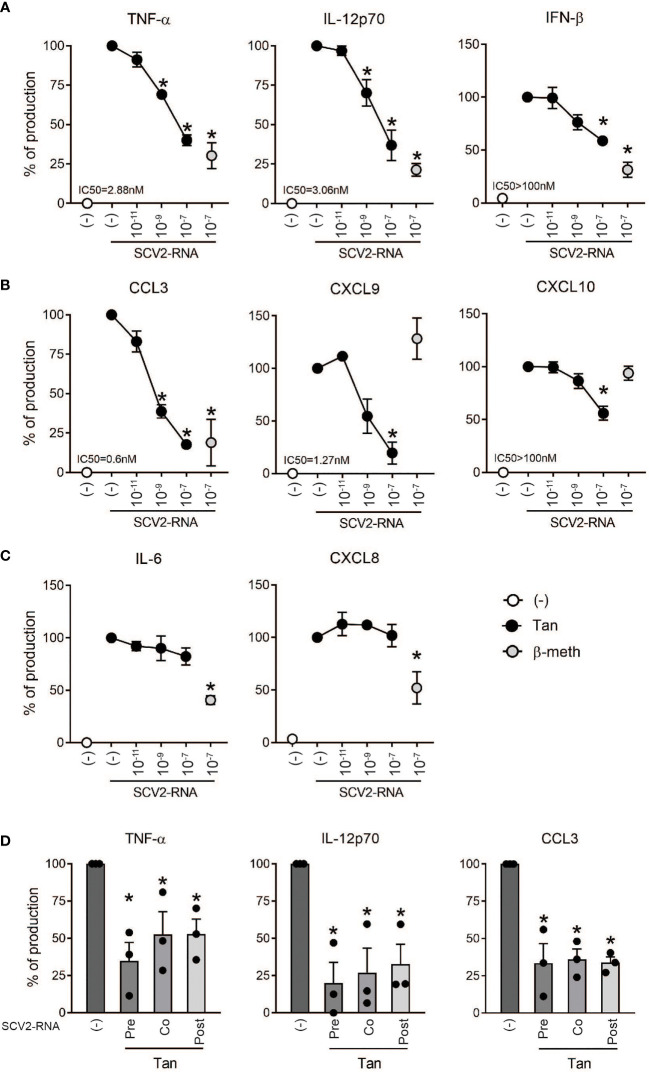
Effect of Tanimilast on cytokine and chemokine secretion by moDCs challenged with SCV2-RNA. **(A–C)** moDCs (2x10^6^/ml) were pre-treated or not (-) with the indicated doses of Tanimilast (Tan) or β-methasone (β-meth) for 1 hour and then stimulated with SCV2-RNA (5 μg/ml) for 24 hours. **(D)** Tanimilast (Tan) was added 1 hour before (Pre), together (Co) and 1 hour after (Post) the stimulation with SCV2-RNA. Cytokine **(A, C, D)** and chemokine **(B, C, D)** production was evaluated by ELISA in cell-free supernatants. Cytokine/chemokine expression was normalized to SCV2-RNA condition (represented as 100%) to control donor-dependent variation. Absolute levels of SCV2-RNA induced cytokines (ng/ml) were: TNF-α=154.79 ± 26.37; IL-6 = 131.66 ± 16.8; IL-12 = 62.53 ± 21.5; IFN-β=0.38 ± 0.2; CCL3 = 42.21 ± 9.79; CXCL9 = 76.1 ± 22.7; CXCL10 = 33 ± 5.8 and CXCL8 = 94.1 ± 10.6. Data are expressed as mean ± SEM (n=3); *P< 0.05 versus SCV2-RNA by one-way ANOVA with Dunnett’s *post-hoc* test. IC50 was calculated by GraphPad Prism nonlin fit log(inhibitor) vs. response.

The effects of Tanimilast on the pro-inflammatory properties of SCV2-moDCs were assessed in terms of cytokine and chemokine regulation. moDCs were pre-treated with Tanimilast (10^-11^, 10^-9^, 10^-7^ M) for 1 hour and then stimulated with an optimal concentration of SCV2-RNA (4). The concentrations of Tanimilast used in this study were previously shown to be effective in moDCs without reducing cell viability [ (15) and data not shown]. β-methasone (10^-7^ M), a glucocorticoid anti-inflammatory drug commonly used to treat overactive inflammation (25), was used as a comparison. **Figure 1A** shows that Tanimilast dose-dependently decreased the production of the pro-inflammatory cytokine TNF-α and of the Th1-polarizing cytokines IL-12 and IFN-β, although with different efficacy. Similarly, also the myelomonocyte-attracting chemokine CCL3 and the Th1-attracting chemokines CXCL9 and CXCL10 were dose-dependently reduced (**Figure 1B**). **Figures 1A, B** show the calculated IC50s that, in most cases, lie in the nanomolar range, a result consistent with previously published data and indicating a high potency of Tanimilast (12, 14, 15). However, in the case of IFN-β and CXCL10, Tanimilast at a concentration of 10^-7^ M (representing the maximal concentration of solubility in our system) could inhibit less than 50% of the secreted cytokine. At 10^-7^ M maximal inhibitory effect on PDE4 is reached (15, 23). Therefore, it is likely that at such concentration also the maximal inhibitory effect of Tanimilast against these two cytokines is reached. Thus, the IC50s could be defined as >100nM. Tanimilast did not inhibit the secretion of the neutrophil-attracting chemokine CXCL8, as well as that of IL-6 (**Figure 1C**). In most cases, β-methasone showed a similar inhibition pattern. At difference with Tanimilast, however β-methasone effectively reduced IL-6 and CXCL8 secretion (60% and 50% reduction respectively, **Figure 1C**), while it did not counteract the induction of CXCL9 and CXCL10 (**Figure 1B**).

Interestingly, significant inhibition of cytokine secretion could also be observed when Tanimilast was administered together and 1 hour after the stimulation with SCV2-RNA, which better mimics a setting in which Tanimilast is used as a therapeutic agent (**Figure 1D**).

These results indicate that both Tanimilast and β-methasone reduce the overall pro-inflammatory potential of SCV2-moDCs. Interestingly, the modulatory pattern of target cytokines differs between the two drugs.

### Tanimilast Does Not Impair the Acquisition of Maturation Markers by SCV2-moDCs

**Figure 2:**
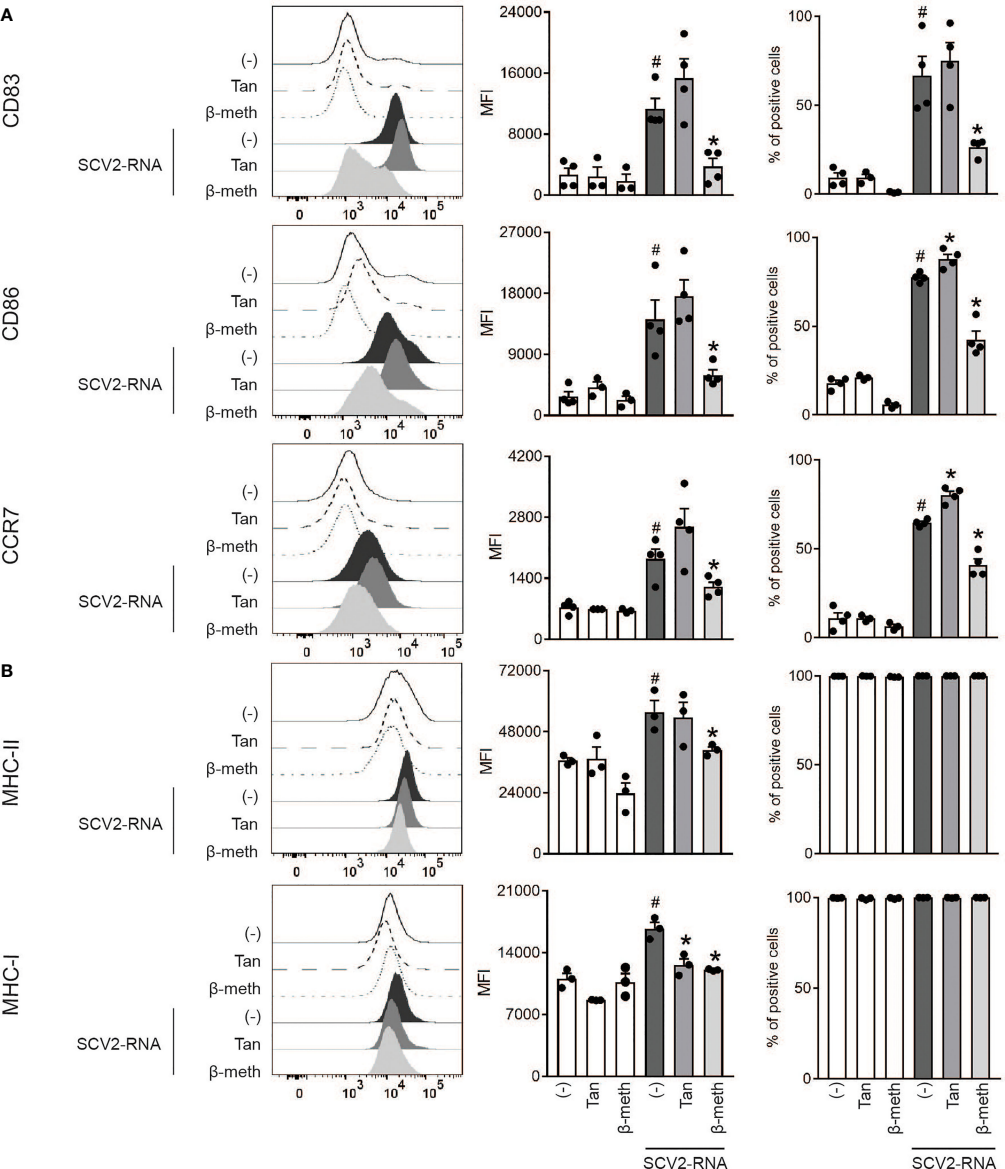
Effect of Tanimilast on moDC phenotypic maturation induced by SCV2-RNA. **(A, B)** moDCs were pre-treated or not (-) with either Tanimilast (Tan) or β-methasone (β-meth) (both at 10^-7^M) for 1 hour and subsequently stimulated or not with SCV2-RNA for 24 hours. The surface expression of activating markers CD83, CD86, CCR7 **(A)** and of antigen presenting molecules MHC-I, MHC-II **(B)** were evaluated by FACS analysis. Data are expressed as representative cytofluorimetric profiles (left panels),as the mean ± SEM (n=3-4) of the Median Fluorescence Intensity (MFI) (center panels) and of the percentage of positive cells (right panels). ^#^P < 0.05 versus (-) and *P < 0.05 versus SCV2-RNA by one-way ANOVA with Dunnett’s *post-hoc* test.

Consistent with our previous findings in LPS-treated moDCs (15), Tanimilast pre-treatment (10^-7^ M) did not restrain the upregulation of the costimulatory molecules CD83 and CD86 and of the lymph-node-homing receptor CCR7 induced by SCV2-RNA (**Figure 2A**, left panels). Indeed, the expression of these markers showed the tendency to be even higher in the presence of Tanimilast both on a per-cell-basis as demonstrated by higher MFI and in terms of % of positive cells (**Figure 2A**, center and right panels). Similarly, Tanimilast pretreatment did not block the upregulation of MHC-II, while consistently reducing the MFI of MHC-I (**Figure 2B** left and center panels). As expected, both these markers were expressed by 100% of the cells in the population in all conditions (**Figure 2B** right panels). By contrast, β-methasone counteracted the SCV2-RNA-dependent upregulation of all these markers. Both drugs did not modify the phenotype of unstimulated moDCs (**Figures 2A, B**, white bars).

Thus, unlike β-methasone, Tanimilast does not grossly impair the phenotypical maturation of moDCs. However, it selectively targets the upregulation of MHC-I, which may result in the modulation of antigen presentation to CD8^+^ T cells.

### Tanimilast Restrains CD8^+^ T Cell Activation by SCV2-moDCs

**Figure 3:**
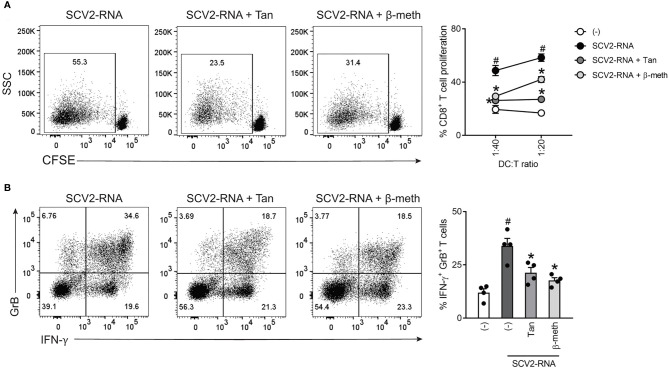
Effect of Tanimilast on CD8^+^ T cell activation by SCV2-moDCs. **(A)** moDCs were treated or not (-) with either Tanimilast (Tan) or β-methasone (β-meth) (both at 10^-7^M) for 1 hour and then stimulated with SCV2-RNA. After 24 hours, moDCs were collected and co-cultured with graded numbers of CFSE-stained allogenic CD8^+^ T cells for 6 days. Alloreactive T cell proliferation was assessed by measuring CellTrace-CFSE dye loss by flow cytometry. Left, dot plot from one representative experiment (1:40 ratio). Right, line graphs from three independent experiments with different DC:T cell ratio. Data are expressed as mean ± SEM (n=3) of the percentage of proliferating CD8^+^ T cells. **(B)** moDCs treated as described in **(A)** were co-cultured with graded numbers of CD8^+^ T cells for 6 days. Intracellular IFN-γ and Granzyme B (GrB) were evaluated by FACS analysis. Left, dot plot from one representative experiment. Right, bar graphs from four independent experiments. Data are expressed as mean ± SEM (n=4) of the percentage of double positive T cells. **(A, B)** ^#^P versus (-) and *P< 0.05 versus SCV2-RNA by *one-way ANOVA with Dunnett’s post-hoc test*.

Based on findings described above, we set up allogeneic co-culture experiments to characterize the CD8^+^ T-cell activating properties of SCV2-moDCs in the presence of Tanimilast. **Figure 3A** shows that, consistent with the observed MHC-I reduction, both Tanimilast and β-methasone impaired CD8^+^ T cell proliferation induced by stimulation with SCV2-moDCs, as assessed by CFSE staining. In addition, both drugs also reduced the percentage of cells producing IFN-γ and Granzyme B, two key effector molecules of activated CD8^+^ T cells (**Figure 3B**).

### SCV2-moDCs Induce a Th2-Skewed CD4^+^ T Cell Response in the Presence of Tanimilast

**Figure 4:**
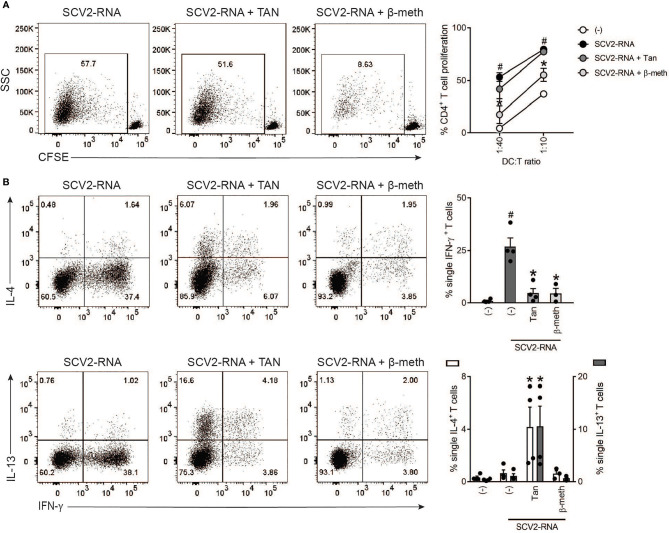
Effect of Tanimilast on CD4^+^ T cell activation by moDCs. **(A)** moDCs were treated or not (-) with Tanimilast (Tan) or β-methasone (β-meth) (both at 10^-7^M) for 1 hour before stimulation with SCV2-RNA. After 24 hours, moDCs were collected and co-cultured with graded numbers of CFSE-stained allogenic CD4^+^ T cells for 6 days. Alloreactive T cell proliferation was assessed by measuring CellTrace-CFSE dye loss by flow cytometry. Left, dot plot from one representative experiment (1:40 ratio). Right, line graphs from four independent experiments with different DC:T cell ratio. Data are expressed as mean ± SEM (n=4) of the percentage of proliferating CD4^+^ T cells. **(B)** Activated moDCs were incubated with graded numbers of T cells for 6 days. Intracellular IFN-γ, IL-4 and IL-13 were evaluated by FACS analysis. Left, dot plot from one representative experiment. Right, bar graphs from four independent experiments. Data are expressed as mean ± SEM (n=3-4) of single IFN- γ- (upper right panel) or single IL-4- (right Y axis) and IL-13- (left Y axis) (lower right panel) producing T cells. **(A, B)** ^#^P< 0.05 versus (-) and *P< 0.05 versus SCV2-RNA by one-way ANOVA with Dunnett’s *post-hoc* test.

The same experiments were performed using naïve CD4^+^ T cells as responders. As expected, based on the lack of MHC-II and costimulatory molecule modulation, Tanimilast did not affect the proliferative response of CD4^+^ T cells induced by SCV2-moDCs (**Figure 4A**). By contrast, β-methasone reverted T cell proliferation almost to basal levels, in accordance with MHC-II downregulation. Next, the effects of Tanimilast on the polarizing properties of SCV2-moDC were assessed by measuring the levels of intracellular cytokines in activated CD4^+^ T cells. We have previously shown that SCV2-RNA induces a prominent Th-1 response (4), which was consistently blocked by both Tanimilast and β-methasone (**Figure 4B**). Interestingly, pre-treatment with Tanimilast, but not with β-methasone, enhanced the development of T cells producing IL-4 and IL-13, which characterize Th2-skewed CD4^+^ effectors (**Figure 4B**). Of note, Tanimilast alone did not induce either IL-4^+^ or IL-13^+^ T cells (data not shown). We also stained for IL-17 production, but this was undetectable in our experimental conditions (data not shown).

Taken together, these results indicate that DCs matured in the presence of Tanimilast fully retain the stimulatory capacity to induce CD4^+^ T cell proliferation while skewing the T helper response toward a Th2 profile. By contrast, the effect of β-methasone results in a general inhibition of CD4^+^ T cell activation, resembling the inhibition observed on CD8^+^ T cells.

### Primary DC Subsets Recapitulate the Effects of Tanimilast Pre-Treatment of moDCs

**Figure 5:**
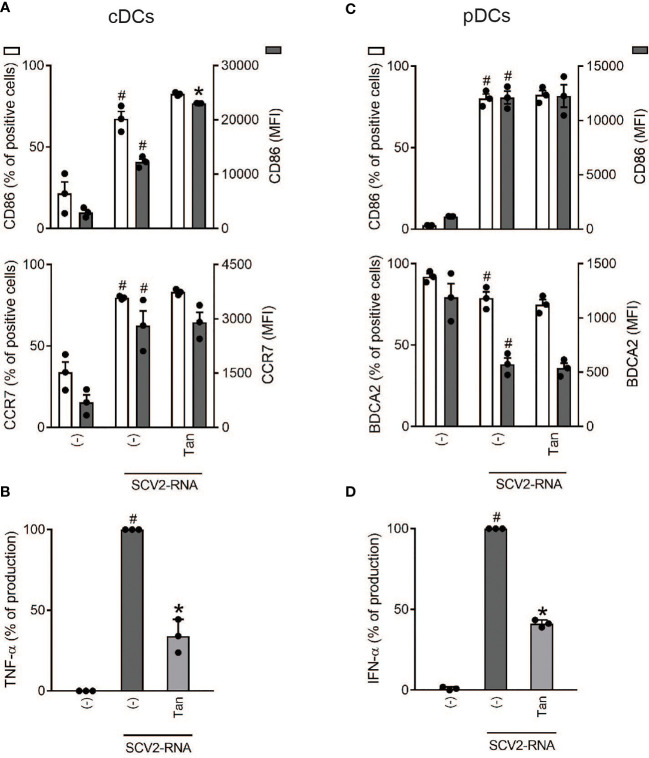
Effect of Tanimilast on primary DC activation by SCV2-RNA. cDCs (2x10^6^/ml) and pDCs (1x10^6^/ml) were pre-treated with Tanimilast (Tan, 10^-7^M) and then stimulated with SCV2-RNA for 24 hours. **(A, C)** The surface expression of CD86, CCR7 and BDCA2 was evaluated by FACS analysis. Data are expressed as the mean ± SEM (n=3) of the percentage of positive cells (left y axis) and of the Median Fluorescence Intensity (MFI) (right y axis). **(B, D)** The production of TNF-α and IFN-α was evaluated by ELISA in cell-free supernatants. Cytokine expression is normalized to SCV2-RNA condition (represented as 100%). Absolute levels of SCV2-RNA induced cytokines (ng/ml) were: TNF-α= 20.92 ± 0.55; IFN-α= 169.36 ± 23.39. Data are expressed as mean ± SEM (n=3). **(A–D)** ^#^P< 0.05 versus (-) and *P< 0.05 versus SCV2-RNA by one-way ANOVA with Dunnett’s *post-hoc* test.

To confirm the results obtained in moDCs also in primary DCs, we immunomagnetically sorted the two main subsets of circulating DCs, namely cDCs and pDCs. Because of the rarity of these cells, only a fixed concentration of Tanimilast was used (10^-7^M). In cDCs, a substantial lack of CD86 and CCR7 modulation by Tanimilast was confirmed, both in terms of percentage of positive cells and of mean fluorescence intensity of the population (**Figure 5A**). By contrast, the production of TNF-α was significantly decreased (**Figure 5B**). Regarding pDCs, though Tanimilast did not interfere with the acquisition of a mature phenotype characterized by the upregulation of CD86 and downregulation of BDCA2 (**Figure 5C**), it decreased IFN-α secretion to 40% (**Figure 5D**).

## Discussion

Tanimilast is a novel inhaled PDE4 inhibitor currently undergoing phase III clinical development for COPD which shows promising pharmacodynamic results associated with a good tolerability and safety profile (14, 24). Tanimilast was previously shown to act as a potent anti-inflammatory agent in several cell-based models (23), including leukocytes derived from asthma (26) and COPD patients (27) and rhinovirus-infected human bronchial epithelial cells (12), as well as in experimental rodent models of pulmonary inflammation (13). In this study, Tanimilast is investigated as an agent capable of modulating the strong inflammatory activation induced by SCV2-RNA in human DCs. Consistent with previous work of our group (15), Tanimilast reduced the secretion of selected, but not all cytokines without affecting the acquisition of a mature phenotype. This is a condition previously defined as “semi-mature DCs”, suited to prevent excessive responses in peripheral tissues (28). Our analysis was conducted in parallel with β-methasone, since corticosteroids are established drugs in the treatment of overactive immune conditions, also undergoing clinical trials for the treatment of COVID-19 (5). Unlike Tanimilast, β-methasone induced a widespread and clear-cut shift from competent to suppressive moDCs. Tanimilast decreased the expression of TNF-α and CXCL10, which are cytokines highly correlated with severity and mortality rate of Covid-19 (29, 30). Additionally, Tanimilast induced a marked reduction in the release of chemokines that amplify the inflammatory and immune response *via* the recruitment of innate cells (e.g. CCL3) or Th1 effector cells (e.g. CXCL9/10). To date, many ongoing trials to test the efficacy of anti-TNF-α or anti-IL-6 drugs in severe COVID-19 have provided conflicting results (31, 32). It is tempting to speculate that Tanimilast may prove beneficial because of its broad modulatory effect on several cytokines, as compared to drugs selectively targeting one specific cytokine.

We observed that Tanimilast, unlike β-methasone, did not inhibit the SCV2-RNA-dependent release of the neutrophil attracting CXCL8, another prognostic marker in COVID-19 (33). However, CXCL8 is produced by many cell types in addition to DCs and was shown to be efficiently blocked by Tanimilast in other experimental settings (15, 34). Thus, our experimental model, by focusing on DCs, may not fully recapitulate the modulation of CXCL8 occurring *in vivo* upon administration of Tanimilast. By contrast, DCs are by far the principal producers of type I IFN, which was decreased by Tanimilast. Because both type I IFNs and pDCs play crucial protective roles in the early phases of SARS-CoV-2 infection (35, 36), the administration of Tanimilast may need to be timely targeted during SARS-CoV-2 infections, especially when tissue damage mostly depends on overwhelming immune activation rather than to viral replication *per se*. This holds true and has been clearly assessed also for corticosteroids, where early addition impairs viral eradication, while late-stage usage reduces symptoms and immune-dysregulation (37).

In the *in vitro* experimental setting utilized in this study, the combined reduction of selected cytokines elicited by Tanimilast, together with the conserved expression of co-stimulatory molecules and MHC class II, skewed the predominantly Th1 polarization of CD4^+^ naïve T cells induced by SCV2-activated DCs (4) towards a Th2-oriented activation, without affecting T cell proliferation. This apparent Th-2 skewing effect of Tanimilast appears to be related to the presence of the SCV2 stimulus. Indeed, we observed that Tanimilast alone induced neither IL-4- nor IL-13-producing T cells. Additionally, Tanimilast is very effective in inhibiting allergen-induced eosinophilia in rats which is Th-2 driven (13). A further evidence of the modulatory effects of Tanimilast on Th-2 driven pulmonary inflammation comes from its ability in reducing the allergen challenge response in asthmatic patients (38). In this regard, the effect of β-methasone was a clear-cut inhibition of phenotypical maturation, CD4^+^T cell proliferation and Th1 blockade, with no observed skewing towards Th2 polarization. We could not evaluate the effects of Tanimilast on Th17 polarization because it was not induced in our experimental setting. However, we demonstrate a reduction in the secretion of crucial Th17-polarizing cytokines such as IL-6 and TNF-α. This is of particular importance, since Th1/Th17 responses have been associated to COVID-19 immunopathogenesis and exacerbation (20, 21). SARS-CoV-2-specific CD4^+^ effector cells generally do not express Th2 traits (39), which could play a protective role as shown by the lower susceptibility and less severe outcomes of COVID-19 in asthmatic and atopic patients (40, 41). Accordingly, IL-13 was shown to reduce viral burden, possibly by downregulating the expression of angiotensin-converting enzyme 2 (ACE2) in airway epithelial cells (42, 43). In addition, M2 macrophage polarization induced by IL-4 and IL-13 fostered tissue repair and resolution of inflammation in ARDS (44). Finally, Th2 cytokines rescue the anti-thrombotic properties of endothelial cells by inhibiting the expression of pyrogen-induced tissue factor (45), which is highly expressed in the lungs of severe COVID-19 patients (46). A number of reports, however, described Th2 signature and eosinophilia in the inflamed areas of lungs in subgroups of severe COVID-19 patients (47). This complex picture reinforces the hypothesis that Tanimilast administration may prove beneficial in blunting the excessive inflammatory response that can occur in severe COVID-19, provided careful patient evaluation and stratification is performed.

Tanimilast reduced the expression of MHC-I molecules. This effect may depend on increased levels of cAMP, reproducing the activation of the cAMP/PKA/ICER pathway previously described to repress MHC-I transcription (48). In addition, PDE4 inhibition by Rolipram was shown to reduce antigen production (and therefore MHC-I expression) by decreasing the activity of the ubiquitin proteasome system in rodent skeletal muscle cells (49). Further research is granted to elucidate if these mechanisms are involved in the block of MHC-I upregulation in Tanimilast-treated moDCs. MHC-I reduction, together with IL-12 blockade, are likely responsible for the observed curtailing of CD8^+^ T cell proliferation and activation, characterized by a decrease of both IFN-γ and Granzyme-B levels. This effect is shared by both Tanimilast and β-methasone. Activated CD8^+^ effector cells play a dual role in SARS-CoV-2 infection, being critical for virus eradication as well as detrimental, when excessive cytotoxic activation results in lung damage, even more lethal than viral replication itself (50). Both hyperactive and exhausted cytotoxic T cells were described in COVID-19 patients, possibly correlating with the course of the illness (51, 52). Indeed, an early immune profile characterized by high expression of interferon stimulated gene and viral load with limited lung damage was shown to precede a later stage with low interferon stimulated gene levels, low viral load and abundant infiltration of activated cytotoxic cells (53). In addition, continual proliferation and overactivation of CD8^+^ T cells observed in severe, late stage COVID-19 were correlated to disease aggravation (54). Thus, the inhibition of CD8^+^ T cells proliferation and activation observed upon Tanimilast treatment may be beneficial to alleviate cytotoxic hyperactivation but might be not relevant, if not contraindicated, in COVID-19 cases displaying an exhausted CD8^+^ T cell phenotype.

Despite this study did not investigate the molecular mechanisms underlying Tanimilast modulation of DC activation by SCV2-RNA, the observed differences in its potency in inhibiting different cytokines suggest a promoter-specific action, rather than a direct perturbation of TLR7/8 signaling and NF-κB activation. This hypothesis is also supported by the observation that genes requiring NF-κB for efficient transcription, such as CCR7 (55) are upregulated in the presence of Tanimilast. A similar mechanism was previously described in LPS-activated moDCs, where we observed that Tanimilast could decrease the recruitment of NF-κB subunits to specific promoters, without affecting its nuclear translocation (15). This could depend on reduced recruitment of NF-κB co-activators, as originally demonstrated for the prototypic PDE4 inhibitor Apremilast (56). Indeed, a promoter specific regulation is very well suited to explain the variegated modulation of DC activation described in this paper.

It remains to be established if immunomodulation by Tanimilast can be observed also when DCs are infected by SARS-CoV-2, instead of being challenged with SCV2-RNA. It was previously shown that Tanimilast could efficiently block rhinovirus-induced cytokines (12). In addition, a recent paper showed that intact SARS-CoV-2 activates innate immune cells *via* TLR7/8, thus reproducing the mechanisms of activation by SCV2-RNA (57). Based on this, it is possible to hypothesize a similar inhibitory effect acting on TLR7/8 downstream pathways, as previously discussed, also in the presence of intact SARS-CoV-2. It should also be considered that, in addition to immunomodulation, Tanimilast may interfere with SARS-CoV-2 infection *via* other mechanisms. For example, Rolipram and Roflumilast were both shown to inhibit viral replication (58, 59). In addition, compounds with properties of PDE4 inhibition were suggested to bind to N-terminal RNA-binding domain of SARS-CoV-2 N-protein, a critical component of the viral replication and genome packaging machinery that may affect viral replication (60, 61). By analogy with other PDE4 inhibitors, it is tempting to speculate that Tanimilast may be helpful in COVID-19 pneumonia not only by regulating the inflammatory balance but also by directly reducing viral replication and load. However, this aspect could not be investigated using our system of moDCs stimulation by SCV2-RNA. Overall, the data presented in this study suggest that the PDE4 inhibitor Tanimilast could be a promising inhaled immunomodulator in the scenario of COVID-19, given its remarkable safety demonstrated in healthy subjects as well as in asthma and COPD patients (14) and its mechanism of action non redundant with corticosteroids. Nevertheless, further studies are needed to evaluate the benefits of this agent in clinical settings. In particular, it will be important to determine the optimal disease stage at which starting Tanimilast administration, with a particular focus on the identification of subgroups of patients (clinical phenotypes) with increased chances of therapeutic success.

## Data Availability Statement

The original contributions presented in the study are included in the article/supplementary material. Further inquiries can be directed to the corresponding author.

## Author Contributions

Conceptualization, DB, VS, and SS. Methodology, VS, TS, DB, FF, and GV. Validation and formal analysis, VS, HON, and TS. Investigation, HON, FS, TS, CG, LT, IB, and MP. Data curation, VS, HON, FS, MP, and IB. Writing—original draft preparation, HON and DB. Writing—review and editing, SS, DB, LT, FF, VS, ADP, GV, and MC. Visualization, HON and IB. Supervision, DB, VS, SS, and FF. Funding acquisition, SS, DB, and VS. All authors have read and agreed to the published version of the manuscript.

## Funding

This research was funded by the Italian Ministry of Health (Bando Ricerca COVID-2020-12371735 to SS), Italian Ministry of the University and Research (MUR-PRIN 20178ALPCM_005 to DB), Associazione Italiana per la Ricerca sul Cancro (IG-20776 to SS), University of Brescia (Fondi Locali 2019 and 2020 to DB and VS). These funders were not involved in the study design, collection, analysis, interpretation of data, the writing of this article or the decision to submit it for publication.

## Conflict of Interest

Authors FF, GV, and MC are employed by Chiesi Farmaceutici S.p.A.

This study received funding from Chiesi Farmaceutici S.p.A. The funder had the following involvement with the study: provided Tanimilast and instructions for its usage, contributed to general research planning and to manuscript review and editing, approved decision to publish.

## Publisher’s Note

All claims expressed in this article are solely those of the authors and do not necessarily represent those of their affiliated organizations, or those of the publisher, the editors and the reviewers. Any product that may be evaluated in this article, or claim that may be made by its manufacturer, is not guaranteed or endorsed by the publisher.
